# Polymer characterization with a fuzzy classification algorithm

**DOI:** 10.1155/S1463924694000209

**Published:** 1994

**Authors:** D. J. Ramsbottom, M. J. Adams, J. Carroll

**Affiliations:** 1 School of Applied Sciences University of Wolverhampton Wulfruna Street WV6 9EP UK; 2 I.C.I. Materials Wilton Middlesbrough Cleveland UK

## Abstract

The classification of polymer samples from their infra-red spectra has been achieved by the application of a fuzzy c-means cluster algorithm. The generation of a fuzzy classifier allows the characterization of samples which are a combination of more than one pure polymer.


Journal of Automatic Chemistry, Vol. 16, No. 5 (September–October 1994), pp. 161–165

### Polymer characterization with a fuzzy classification algorithm

**D. J. Ramsbottom, M. J. Adams***

*School of Applied Sciences, University of Wolverhampton, Wulfruna Street, WV6 9EP, UK*

**and J. Carroll**

*I.C.I. Materials, Wilton, Middlesbrough, Cleveland, UK*

*The classification of polymer samples from their infra-red spectra has been achieved by the application of a fuzzy* c*-means cluster algorithm. The generation of a fuzzy classifier allows the characterization of samples which are a combination of more than one pure polymer.*

### Introduction

The widespread use of personal computer systems in the analytical laboratory, and the processing power they offer, has supported the increasing interest in the application of chemometrics and multivariate analysis. The development and use of pattern recognition, PR, techniques for classifying known samples and identifying new samples has received a great deal of attention in the literature. A wide range of PR algorithms have been reported; these methods are usually categorized as 'supervised' PR (in which a training set of previously identified samples are analysed in order to determine some discriminant function which allows subsequent samples to be classified), or 'unsupervised PR' (commonly referred to as 'cluster analysis'). The primary aim of a cluster analysis is to seek and identify implicit structure in unclassified sets of data. Numerous texts and reviews are available describing the principles and applications of such data manipulation techniques [1–4].

While hierarchical cluster analysis is one of the most popular PR techniques in science, because of the familiar dendrogram representation of association between objects, the use of this graphic display can produce considerable distortion in the data representation and the actual display obtained is dependent on the method employed. Non-hierarchical techniques do not directly distort the data, but their graphic representation is less obvious and the results may not be simple to interpret. The aim of the present study was to investigate the implementation of a non-hierarchical clustering algorithm, based on fuzzy pattern recognition, and its application to the classification of polymer films by means of their infra-red spectra.

The theory of fuzzy sets, in a form suitable for computerized implementation and as a formal branch of mathematics, was first proposed by Zadeh [5]. The theory extends crisp set theory, in which an item either belongs or does not belong to a set, to define an object's membership function, or degree of belonging to a set. Such membership functions define a transition from wholly belonging to not belonging; they are continuous, monotonically increasing or decreasing functions. Fuzzy set theory is currently receiving considerable attention in a wide range of laboratory automation and data processing tasks as it provides a means of expressing uncertainty or imprecise (fuzzy) data in a computer readable format. An overview of fuzzy set theory and potential applications in analytical chemistry has been presented by Otto [6].

The concept of a membership function is directly applicable to pattern recognition and cluster analysis as it provides a numerical value expressing similarity between items rather than the binary yes/no classifier's associated with conventional binary discrimination functions. Fuzzy pattern recognition algorithms have been extensively reviewed by Bezdek [1]—the algorithm implemented in this study is the fuzzy $c$-means method. The application of the algorithm to synthetic artificial data and to the classification of a selection of IR spectra of polymers is demonstrated.

### Fuzzy classification algorithm

Cluster analysis seeks to achieve classification of a group of samples or objects according to the similarity of features measured on each object. Clearly defined clusters will be characterized by member objects being similar or close in the multidimensional feature space, and well separated from other clusters. A common measure of similarity, and that used in the present study, is the Euclidean distance between objects. The fuzzy $c$-means clustering technique seeks to minimize the within-cluster squared Euclidean distance between the objects and the centre of each cluster. The number of clusters, $c$, is pre-selected by the user. The algorithm to generate the $c$-partitions and, hence, the $c$-clusters was proposed initially by Bezdek *et al.* [3] and the computerized implementation has been discussed by Zadeh [7]. The degree or extent to which an object, $i$, belongs to a specific cluster, $c$ is referred to as that object's membership function ($\mu_{ci}$). From a preselected number of clusters and a randomly generated initial fuzzy partition of the objects such that there are no empty clusters, and the membership functions for an object with respect to each cluster sum to unity, the algorithm proceeds iteratively. First, weighted means representing the cluster centres are calculated. From these centre values, new membership functions for each object with respect to each cluster are calculated, and new fuzzy partitions of the sample space are derived. This process continues with new cluster means and new membership functions until the total change in membership functions is considered insignificant and the cluster populations remain unchanged. The mathematical basis of the algorithm and

* *Correspondence to Dr Adams.*

0142-0453/94 $10.00 © 1994 Taylor & Francis Ltd.

proposed stopping conditions have been described by Bezdek *et al.* [3] and Zadeh [7].

In this study the program was implemented in C language on a 386-based microcomputer. The results comprised a list of $c$-cluster centres, and a value denoting the object's degree of membership to each cluster.

### Test data

Typical results obtained with the fuzzy $c$-means algorithm can be illustrated using the 'butterfly' data set, which is bi-variate and two-dimensional data—see figure 1($a$). If two clusters are selected, then the membership of each observation to its nearest cluster centre ($\mu$) is as shown. The selection of $c$, the number of clusters into which the data are to be partitioned, is chosen by the user. As with traditional clustering techniques, there are no formal rules to dictate what is optimal or best. Interpreting clusters is largely subjective, meaningful clusters can often be obtained for several values of $c$. Several objective functions to assess cluster validity have been reviewed by Bezdek [1]; a particularly useful one, which is simple to implement, is the partition coefficient defined by:

$$F(\mu; c) = 1/n \sum_{k=1}^{n} \sum_{i=1}^{c} \mu_{i,k}^2$$

where $\mu_{ik}$ is the degree of membership of the object to the cluster, $i$, and $n$ is the total number of objects.

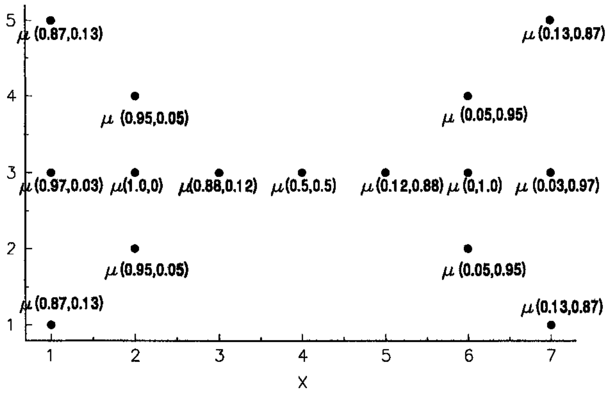

*Figure 1(a). The butterfly data set with membership values μ (A, B), where A is the membership to cluster 1 and B is the membership to cluster 2.*

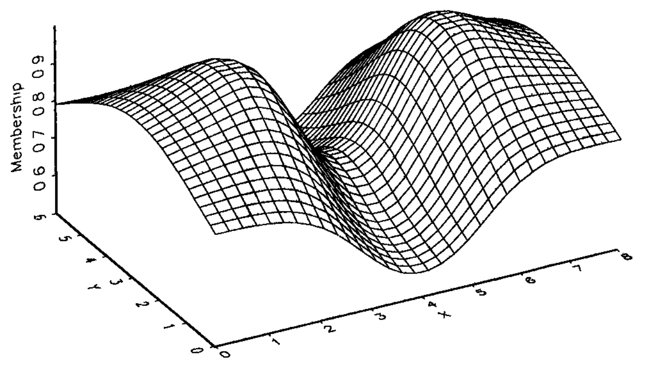

*Figure 1(b). The membership profile to 2 clusters of the butterfly data.*

*Table 1. The partition coefficients for 2, 3, 4 and 5 clusters for the butterfly data, showing two clusters to be an optimum.*

| Clusters | Partition coefficient |
|---|---|
| 2 | 0·843 |
| 3 | 0·685 |
| 4 | 0·592 |
| 5 | 0·609 |

The computed value for $F(\mu; c)$ depends on all observations and maximizing this function can produce the most valid clustering. The butterfly data were examined with values for $c = 2$, 3, 4 and 5, and table 1 shows the partition coefficients calculated for each value. As to be expected $c = 2$ provides an optimal clustering as given by the maximum value for $F(\mu; c)$, as well as the most intuitively meaningful.

Once a satisfactory clustering and partition is achieved, a membership function surface can be created using the original data as a training set, as illustrated in figure 1($b$), for $c = 2$. New, unclassified samples can be subsequently classified by interpolation on the surface.

The subjective nature of cluster analysis can be better appreciated by reference to a more diffuse data set. Figure 2 illustrates the scatter plot of a bi-variate data set [8]. The natural clustering patterns are not so obvious or unambiguous as with the butterfly data. Using fuzzy $c$-means, the results for $c = 2$, 3, 4 and 5 are illustrated in figure 3, together with the computed partition coefficients. High values of $F(\mu; c)$ are obtained for each value of $c$ tested, a result in agreement with visual interpretation of the data since each group of partitions has significance, depending on the context of the interpretation. It is of particular interest and value to note that, unlike hard clustering techniques, fuzzy clustering does not force an object into one or other cluster but can allow it to belong equally to two, or more, groups.

### Characterization of polymers

The use and application of the fuzzy $c$-means algorithm was investigated further using infra-red spectral data and examining the ability of the technique to classify spectra.

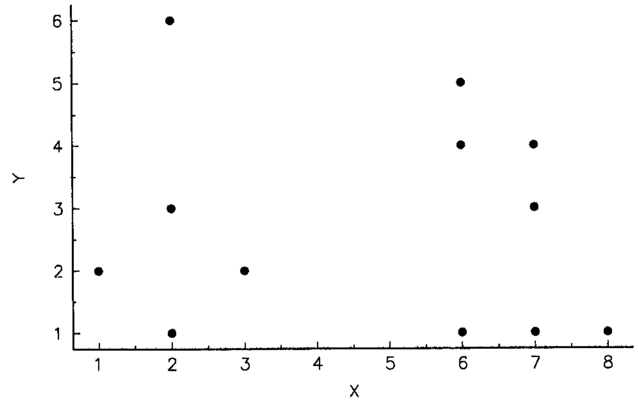

*Figure 2. The test data employed by Zupan [8].*

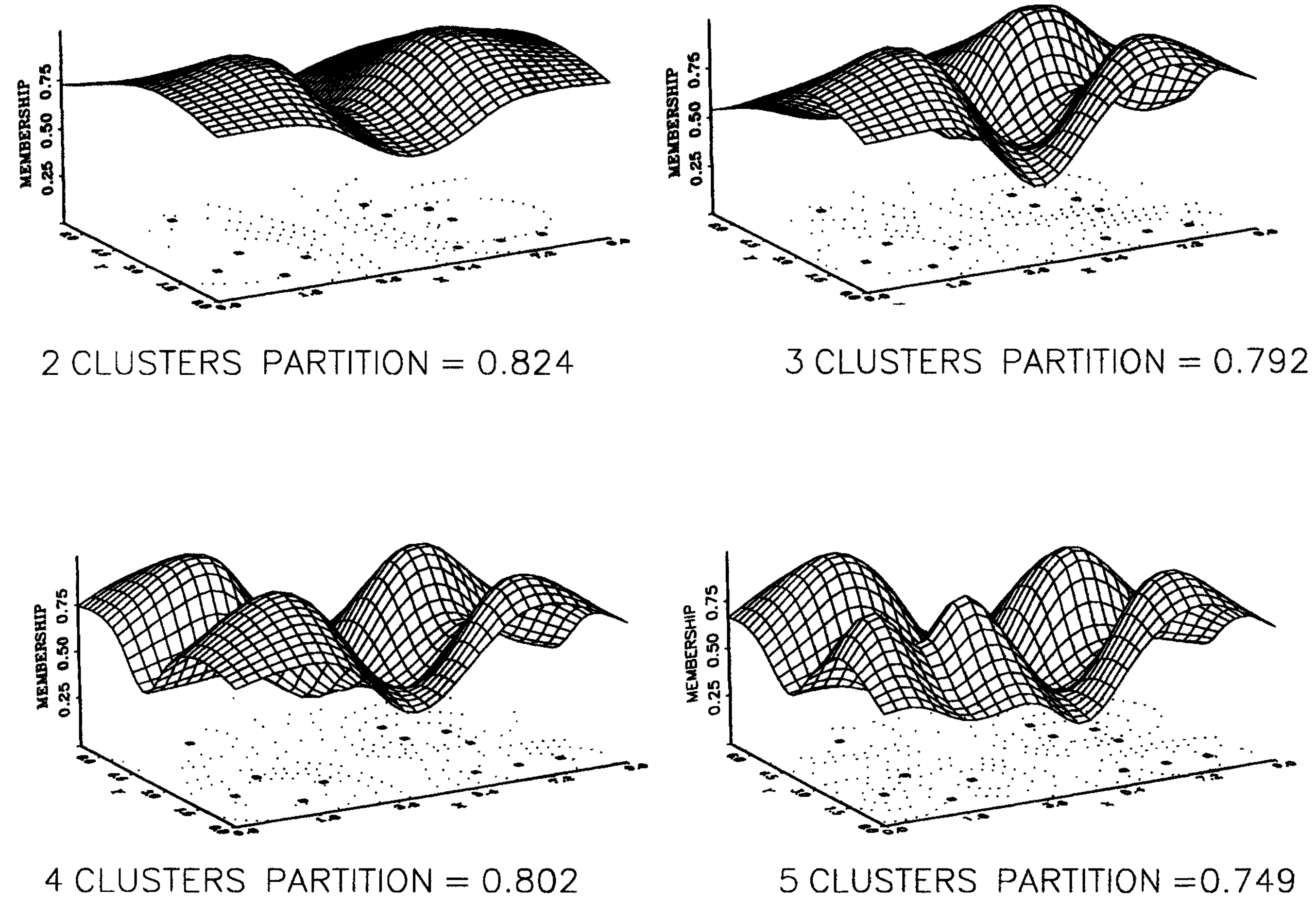

*Figure 3. Membership profiles and partitions for 2, 3, 4 and 5 clusters of the test data from figure 2.*

Polymer samples for characterization were acquired from a number of sources, including a commercial polymer identification kit [9] and an industrial polymer analysis laboratory (ICI Materials Research Centre, Middlesbrough, Cleveland, UK). Several homopolymers were chosen with differing copolymers, copolymer levels, additives and additive levels. These were prepared for analysis as thin films, using standard methods of pressing under hot plates or casting from solution.

*Table 2. The variance and cumulative variance from a principle component analysis ofthe polymer spectra.*

| Principal components | Variance | % cumulative variance |
|---|---|---|
| 1 | 53·46 | 53·46 |
| 2 | 25·20 | 78·66 |
| 3 | 12·60 | 91·26 |
| 4 | 3·60 | 94·86 |
| 5 | 2·06 | 96·92 |

### Data acquisition and pre-processing

The infra-red spectra were acquired on a Philips PU9624 FTIR spectrometer from 4000 to 666 $cm^{-1}$ (2·5–15 microns) at a resolution of 4 $cm^{-1}$, to provide absorbance spectra containing 867 points each. All spectra were normalized on the most intense absorption band to reduce film thickness effects. As infra-red spectral data are highly correlated, data reduction was carried out in two steps to decrease the number of variables required to represent the spectra. First, the spectra were reduced to 216 values by four-point averaging, and then a principal component analysis (PCA) was performed.

It can be seen from table 2 that the first three principal components account for more than 91% of the total variance within the spectral data. A scatter plot of the polymer data loaded onto these components is shown in figure 4. It is evident that these three components provide for effective and clear separation between groups of samples. The first component, PC1, forms a partition between acrylic polymers and others. PC2 forms two partitions, nylon and PVC polymers are separated from styrene and acrylic polymers and PC3 provides for a partition between styrene and the others.

Examination of the principal component loading spectra (figure 5) highlights the weighting given to each data point in the spectra. Where each partition is formed, the majority of bands in the corresponding spectra received either a strong positive or negative weighting. Where PC2 produces two partitions it can be seen that the bands in the nylon spectra receive positive weighting and the bands in the PVC spectra receive negative weighting. Where the bands in a spectrum do not conflict, as in the carbonyl band at 1700 $cm^{-1}$ for acrylic in figure 5(*a*), the

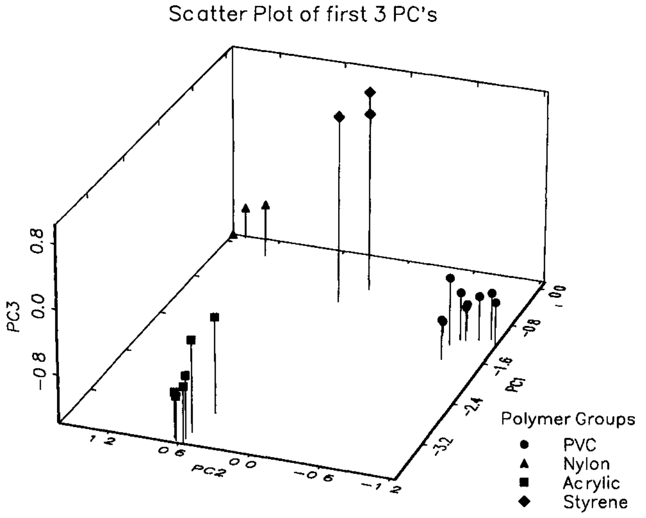

*Figure 4. A scatter plot of the first three principal components of the four polymer groups.*

weightings are high. However, where common bands exist, for example, about 3000 $cm^{-1}$ in figures 5(*b*) and 5(*c*), the weighings are small. As there are subtle differences between spectra in each group, only the common base polymer bands receive weightings.

The occurrence of copolymers and blends of polymers is quite common in plastics, and the application of fuzzy *c*-means methods to cluster the data set has an intuitive appeal as unknown samples may indeed be a mixture of two groups, and may therefore have a relatively high membership to the cluster of each pure polymer group.

Subsequent characterization of an unknown sample requires the same data reduction steps to be carried out as for the test samples, i.e. reduction to 216 points and application of the calculated principal component vectors to give three principal components. These are used to calculate the membership of the unknown sample to each

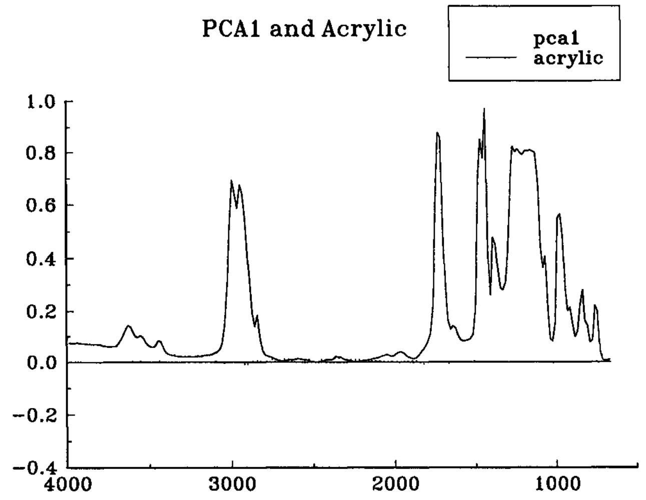

*Figure 5(a). The weightings given by PC1 plotted with an infra-red spectrum of an acrylic sample. The major bands in the spectrum receive negative weightings, causing the samples to be partitioned into two groups.*

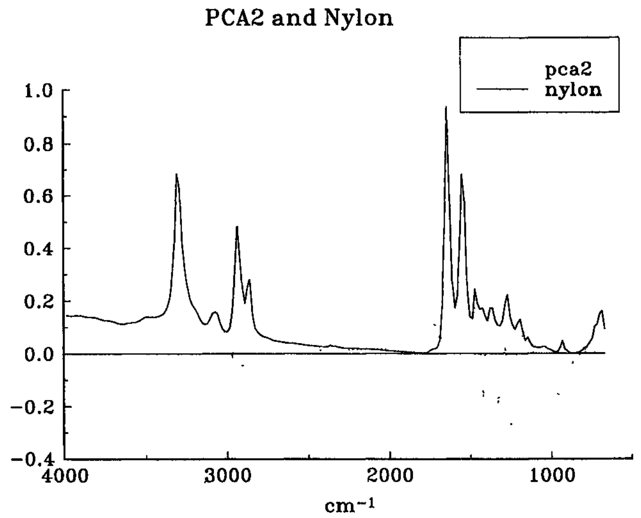

*Figure 5(b). The weightings given by PC2 plotted with an infra-red spectrum of nylon, showing the nylon bands receiving positive weightings.*

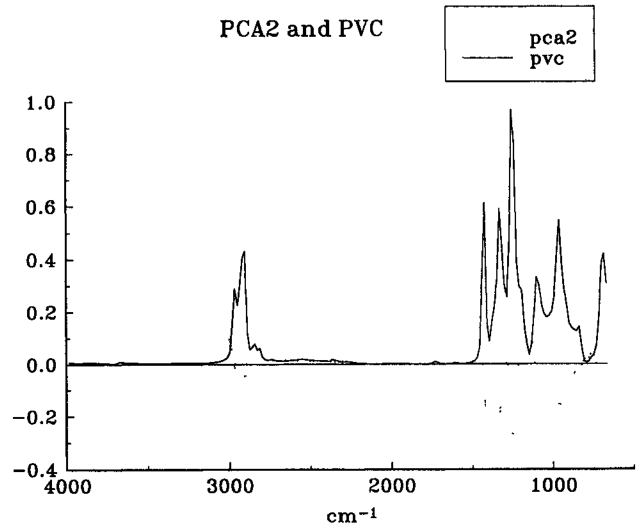

*Figure 5(c). The weightings given by PC2 plotted with an infra-red spectrum of PVC, showing the PVC bands receiving negative weightings.*

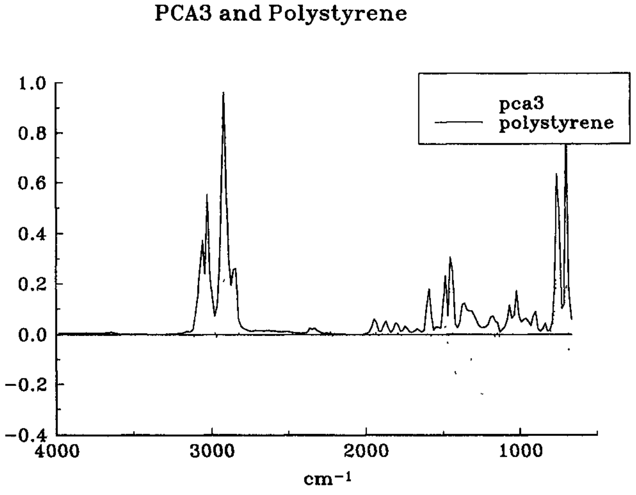

*Figure 5(d). The weightings given by PC3 plotted with an infra-red spectrum of polystyrene, showing the polystyrene bands receiving positive weightings.*

*Table 3. The membership values for a series of plastic samples, A–F. Where A = acrylic; B = polystyrene; C = polystyrene; D = nylon; E = PVC; and F = acrylic/styrene blend.*

| Sample code | Standard polymers | | | | |
|---|---|---|---|---|---|
| | Acrylic | Styrene | PVC | Nylon | Actual main polymer |
| A | 0·40 | 0·19 | 0·25 | 0·16 | Acrylic |
| B | 0·005 | 0·97 | 0·015 | 0·01 | Styrene |
| C | 0·01 | 0·97 | 0·015 | 0·005 | Styrene |
| D | 0·032 | 0·155 | 0·089 | 0·724 | Nylon |
| E | 0·18 | 0·30 | 0·33 | 0·18 | PVC |
| F | 0·33 | 0·25 | 0·24 | 0·18 | Acrylic and styrene |

cluster. The test plastics examined were ground to powder and their spectra obtained as compressed KBr discs. This produced spectra with lower signal-to-noise ratios than spectra from thin films and sloping base-lines due to scattering and diffraction effects. However, with base-line correction, it is evident from the results shown in table 3 that correct identification and classification of each sample was possible.

### Conclusion

A pattern recognition program based on the fuzzy *c*-means algorithm has been implemented, and evaluated for artificial data sets and data obtained from an infra-red analysis of a range of polymer samples. The results obtained illustrate that the method is capable of providing meaningful clustering and classification, without the problems associated with producing traditional binary classification decisions.

The calculations, and generation, of membership function values as obtained from the algorithm discussed above, are of interest not only for their intrinsic value in direct classification, but also for their potential use in broader based applications. For example the development of expert systems for the interpretation of analytical data has indicated that fuzzy logic represents an excellent means of developing and propagating uncertainty within such systems [10]. A serious problem associated with integrating uncertainty in expert systems lies with generating numerical values of uncertainty from analytical data. The fuzzy *c*-means algorithm provides these values and the algorithm can be linked directly to an expert system shell. As applications of artificial intelligence continue to grow in analytical science, the effect of uncertainty and its measurement and integration into data analysis schemes will become more important.

### Acknowledgements

The authors wish to acknowledge I.C.I. Wilton, UK and Unicam Analytical Systems, Cambridge, UK for samples and their support in this project.

### References

1. Bezdek, J. C., *Pattern Recognition with Fuzzy Objective Function Algorithm* (Plenum Press, New York, 1987).
2. Senn, P., *Kemija u Industriji*, **39** (1990), 177.
3. Bezdek, J. C., Ehrlich, R. and Full, W., *Computers and Geosciences*, **10** (1984), 191.
4. Miyamoto, S., *Fuzzy Sets in Information Retrieval and Cluster Analysis* (Kluwer Academic Publishers, The Netherlands, 1990).
5. Zadeh, L. A., *Information and Control*, **8** (1965), 338.
6. Otto, M., *Chemometrics and Intelligent Laboratory Systems*, **4** (1988), 101.
7. Zadeh, L. A., in *Classification and Clustering*, edited by Van Ryzin, J. (Academic Press, New York, 1977).
8. Zupan, J., *Clustering of Large Data Sets* (Wiley and Sons, Chichester, 1982).
9. The Resin Kit Company, 1112 River St., PO Box 509, Woonsocket, RI 02895.
10. Ramsbottom, D. J., Adams, M. J., Sumiga, J. and Carroll, J., *Chemometrics and Intelligent Laboratory Systems*, **19** (1993), 53.
11. Perkins, J. H., Hasenoehrl, E. J. and Griffiths, P. R., *Analytical Chemistry*, **63** (1991), 1747.

